# Newer Perspectives of Mechanisms for Euglycemic Diabetic Ketoacidosis

**DOI:** 10.1155/2018/7074868

**Published:** 2018-10-02

**Authors:** Xiaofang Yu, Saifei Zhang, Long Zhang

**Affiliations:** Department of Endocrinology, Ningbo Medical Center Lihuili Hospital, Ningbo 315040, China

## Abstract

Euglycemic diabetic ketoacidosis (EDKA) was considered a rare condition with its specific definition and precipitating factors. However, with the wide use of sodium glucose cotransporter 2 (SGLT-2) inhibitors, the newest class of antidiabetic agents, EDKA has come back into the spotlight. Relevant cases are increasingly being reported along with insights into the mechanism of EDKA. It seems increasingly clear that EDKA is more common than we used to believe. The SGLT-2 inhibitor-associated EDKA also indicates a necessary review of our previous understanding of “diabetic” ketoacidosis, since the SGLT-2 inhibitor predisposes patients to DKA in a “starvation” way. Actually, there are growing reports about starvation-induced ketoacidosis as well. The previously “exclusive” nomenclature and cognition of these entities need to be reexamined. That the hormonal interactions in DKA may differ from the severity of insulin deficiency also may have served in the scenario of EDKA. The SGLT-2 inhibitors are newly approved in China. The main purpose of this work is to have a better understanding of the situation and update our knowledge with a focus on the pathogenesis of EDKA.

## 1. Introduction

The newest class of antidiabetic agent SGLT-2 inhibitor is widely used with its confirmative effects on lowering blood glucose, blood pressure, and uric acid and favorable cardio-reno outcomes [1–3]. Along with it is the issue of possible adverse events of DKA [4–6]. Most of the reported SGLT-2 inhibitor-associated DKA are euglycemic DKA (EDKA) [4–6]. So far, SGLT-2 inhibitors are becoming a representative aetiology of EDKA and have fueled a surge of interest in revisiting this “old” topic.

Due to the keener clinical perception of this entity, more EDKA cases are reported [7–13]. It is becoming increasingly clearer that EDKA is not so rare as we used to believe. It is possible that many cases were undiagnosed or misdiagnosed. Two SGLT-2 inhibitors, dapagliflozin and empagliflozin, are newly approved by the Chinese Food and Drug Administration. A better understanding of the underlying mechanism will help optimize clinical application of this new star medication.

## 2. Case Representation

We reviewed all 156 DKA admissions in our medical center during the past 4 years and identified 4 cases of EDKA with an incidence of 2.6%, which would shed some light on the frequency of EDKA in real clinical work before the application of SGLT-2 inhibitors. The 4 cases of EDKA are briefly described as follows:

Case patient #1 was a 20-year-old female with type 1 diabetes on a basal-bolus insulin regimen. She had a sore throat and malaise for the previous 3 days and was self-diagnosed as having “flu” and treated through drinking more water. Since she lost her appetite and ate little, she had skipped premeal injection of insulin lispro for 2 days but continued to inject insulin glargine at a reduced dose (from 15 U to 10 U). Physical examination revealed a moderate swelling in her bilateral tonsils with no indication of purulence, and examinations of the lungs, the heart, and the abdomen were normal. The vital signs were within normal. Her point-of-care blood glucose was 10.4 mmol/l. Considering her frank type 1 diabetes history, the ER physician ordered an arterial blood gas analysis (ABG) which showed a pH of 7.23 and an HCO_3_^−^ of 14.9 mmol/l. Along with a positive urinalysis, a diagnosis of DKA was made. Treatment of hydration and small-dose intravenous insulin infusion were administered, along with 5% dextrose to maintain her blood glucose at 7.8~14.1 mmol/l. The episode of acidosis was completely resolved on the next day.

Case patient #2 was a 54-year-old female with a known history of schizophrenia treated with clozapine and sertraline hydrochloride. She had developed anorexia, polyuria, and polydipsia for a month and was escorted to the ER because of nausea, vomiting, and abdominal pain for 2 days. On presentation, she had a slow response but well oriented. Physical examination showed tachycardia and mild tenderness below the umbilicus without muscle guarding. The vital signs were within normal. Routine point-of-care blood glucose testing was 9.0 mmol/l. The blood work showed the following: white blood cell count (WBC) (10 × 10^9^/l), neutrophils (6.5 × 10^9^/l), amylase (168 U/l), Na^+^ (146 mmol/l), K^+^ (2.9 mmol/l), and Cl (96 mmol/l). A CT scan abdomen was ordered. In the meantime, the patient was given 0.9% saline transfusion together with antibiotics and PPI (proton pump inhibitor). In the following hour, the patient was restless and developed dyspnea. An instant ABG analysis showed a pH of 7.15, a P_CO2_ of 23, an HCO_3_^−^ of 13.9 mmol/l, a plasma lactic acid of 0.6 mmol/l, a Na of 143 mmol/l, a K of 2.5 mmol/l, and a glucose of 10.2 mmol/l. Urine analysis: keton bodies (+++), glucose (++). Abdominal CT got back negative. She was then admitted to the hospital and treated with DKA. 5%~10% dextrose was administered along with intravenous insulin infusion. The metabolic acidosis was resolved on the second day, and her plasma amylase readily decreased. Further tests showed an HbA1c of 9.4%, a negative GAD antibody, and a fasting triglyceride of 1.71 mmol/l. She was diagnosed with type 2 diabetes mellitus. There was no family history of diabetes. Her weight gain during the past two years and medical history of schizophrenia and clozapine therapy were considered risk factors. The rest of her hospitalization was eventless, and she was discharged on metformin and gliclazide.

Case patient #3 was a 30-year-old woman with 1 year of type 2 diabetes mellitus. She was pregnant for 28 weeks (G2P0, with a miscarriage 4 years ago). She was previously on the dual therapy of metformin/sitagliptin and switched to premixed human insulin formula (Humulin 70/30R) after she got pregnant. For this very visit, she was scheduled for the second laser treatment for her eyes. She was feeling well, but the random urine analysis revealed 4+ of the ketone body. She also admitted to have repeated ketonuria at 2+~3+ during her obstetric follow-up. Her urine ketone remained 3+ after admission while the random blood glucose was 6.7 mmol/l. The ABG analysis revealed the following: pH (7.31), pCO_2_ (28 mmHg), HCO_3_^−^ (19.2 mmol/l), Na (141 mmol/l), K (4.2 mmol/l), Cl (100.0 mmol/l), blood *β*-hydroxybutyric acid (4.2 mmol/l), and HbA1c (6.7%). Consistent ketosis with anion gap metabolic acidosis was confirmed. Since she did not have trouble eating, oral hydration was implemented, as well as customized diet suggestions. Meanwhile, her insulin therapy was switched to the basal-bolus regimen with premeal insulin aspart (6 U) and bedtime insulin detemir (8 U). Her blood glucose was under control, and ABG results improved. The ABG analysis on the third day showed a pH of 7.36, a pCO_2_ of 29 mmHg, and an HCO_3_^−^ of 22 mmol/l. Urinalysis showed a 1+ of the ketone body. She got her second laser therapy during the hospital stay and returned to the local obstetrics center for follow-up. She gave birth to a 3.3 kg healthy boy by cesarean on the 36th week.

Case patient #4 was a 54-year-old male with type 2 diabetes mellitus for 20 years. He had been on CSII (continuous subcutaneous insulin infusion) therapy for 8 years and also started liraglutide (1.2 mg/day) 3 years ago. He was sent to the ER because of melena for 2 days and hematemesis once. He was admitted to the gastroenterology department with upper gastrointestinal hemorrhage. He admitted to have alcohol consumption right before this episode. Standard fasting and PPI therapy were started along with fluid replacement (mostly isotonic saline). The CSII and liraglutide therapy were stopped since his blood glucose remained low (5.2~12.9 mmol/l). On the third day, the patient developed nausea and vomiting and had labored breathing. The cardiac serum markers remain normal, and his ABG analysis revealed metabolic acidosis: pH (7.25), pCO_2_ (25 mmHg), HCO_3_^−^ (15.7 mmol/l), Na^+^ (142 mmol/L), K^+^ (3.0 mmol/l), and Cl (100.2 mmol/l). The plasma blood glucose was 9.7 mmol/l, and blood *β*-hydroxybutyric acid was 3.1 mmol/l with a urine ketone of 3+. The consultant endocrinologist gave the diagnosis of EDKA and suggested intravenous insulin infusion along with dextrose to keep the blood glucose around 10 mmol/l. His symptoms got relieved. Acidosis was corrected within 6 hours. The patient then resumed continuous basal insulin infusion via his insulin pump. The gastroscopy revealed a duodenal bulbar ulcer. The rest of his hospitalization was uneventful.

The clinical features of the 4 patients are summarized in Table 1.

## 3. Discussion

We identified 4 patients as having EDKA in 156 DKA admissions. Three of them had type 2 diabetes, and only one patient had type 1 diabetes. Thanks to the physicians' clinical vigilance, all patients got timely diagnosis and treatment before their conditions critically worsened. Our medical center does not usually treat pediatric patients, which explains the less type 1 patients we have. The same goes with diabetic pregnancies since we do not have an obstetric clinic. Even so, the incidence of EDKA is surprisingly high, indicating that it is a more common manifestation in DKA. With the advent of SGLT-2 inhibitors in clinical practice, a better understanding of its pathogenesis is necessary for the recognition of susceptible patients and conditions.

EDKA was first described by Munro et al. as DKA episodes with blood glucose < 300 mg/dl and plasma bicarbonate ≦ 10 mmol/l [14]. Jenkins et al. reported 23 EDKA out of 722 DKA episodes (3.2%) in 1993 based on the same criteria as what Munro et al. adopted [15]. They proposed blood glucose < 10 mmol/l as the glucose criterion for true EDKA and reported an incidence of 0.8~1.1% (depending on the plasma bicarbonate criteria ≤ 10 mmol/l or ≤15 mmol/l) [15]. Currently, the blood glucose criterion for EDKA is <200 mg/dl (11.1 mmol/l) [16].

EDKA was used to be taken as a rare condition happening predominantly in type 1 diabetes. As the truth, all the patients Munro et al. [14] and Jenkins et al. (except for one old patient with myocardial infarction) [15] reported in their studies had insulin-dependent diabetes. The intrinsic insulin deficiency in these patients were the self-evident setting for the EDKA episodes, with decreased carbohydrate and maintenance (in many cases with increased doses) of insulin therapy responsible for the relatively low blood glucose [14, 15]. Less dehydration and continuing urinal loss of glucose were also considered possible contributors [15]. So it is the historical meaning of true euglycemic “diabetic” ketoacidosis.

But it is only a small part in the whole picture of EDKA, so is the assumed mechanism. Even in this subset of classical cases of EDKA [14], there was a debate from the beginning whether the “normal” glucose in EDKA is partly derived from decreased endogenous glucose production via gluconeogenesis or from increased urinal loss [14, 17]. The question might have remained largely unresolved [18–21]. So far, it is clear that hepatic glucose production rates vary widely among DKA episodes from decreased and normal to elevated rates [18–21], so is the endogenous insulin concentration in DKA episodes [22]. Different precipitating conditions like the severity of fasting or dehydration may hold responsible for the variety [18]. But the significance of the variety in particular patients remains obscure.

**Figure 1:**
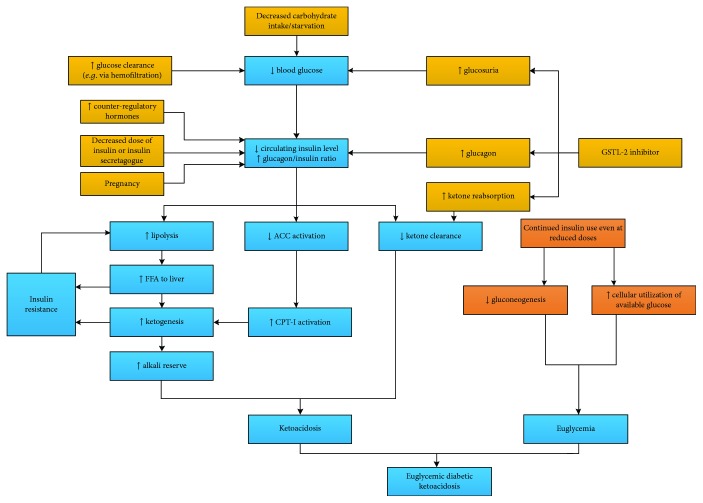
Possible pathogenesis of EDKA. ACC: acetyl coenzyme A carboxylase; CPT-I: carnitine palmitoyltransferase-I; FFA: free fatty acid.

The current mainstream approaches to the mechanism of EDKA are essentially the same as the initial assumptions made by several authors [14–16, 19]. Decreased carbohydrate intake causes insulinopenia and increased glucagon. Increased glucagon/insulin ratio further promotes lipolysis and ketogenesis. Meanwhile, carbohydrate deficit and continued insulin treatment facilitate “euglycemia” (Figure 1). A well-known aetiology of EDKA includes pregnancy [16, 23], glycogen storage disorders [13, 24], diet restriction/starvation [12, 13], and alcohol and SGLT-2 inhibition [7, 16]. However, the mechanism is not without doubt. For one thing, it is ambiguous about the insulin deficiency and insulin compensation. It is already well known that the insulin concentration required to suppress lipolysis is far lower than what is needed to promote glucose utilization [25–27], although it is also true that the hypoglycemic activity of insulin does not have the “threshold” phenomenon while its antilipolytic and antiketotic activities do [27]. For another, it is hard to say what actually triggers EDKA.

**Figure 2:**
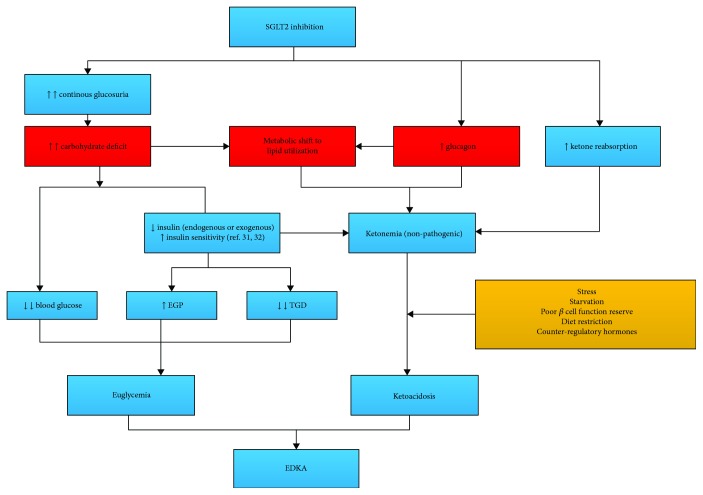
Pathogenesis of SGLT-2 inhibitor-associated EDKA. EGP: endogenous glucose production; TGD: tissue glucose disposal.

Thus far, studies on the pathogenesis of SGLT-2 inhibitor-associated EDKA have cleared some things up about EDKA [28–33]. By competitive inhibition of SGLT-2 at the proximal convoluted tubule, SGLT-2 inhibitors block the reabsorption of 30~50% of filtered glucose from the primary urine [28, 29]. The hypoglycemic effect of this “carbohydrate deficit” is only partly offset by the increased endogenous glucose production (EGP) via gluconeogenesis and the decreased tissue glucose disposal (TGD) [31, 32]. There was a metabolic shift from glucose utilization to lipid utilization, just as what happens in starvation [33]. The lower blood glucose causes a decrease in circulating insulin and an increase in glucagon concentration. The SGLT-2 inhibitor per se is a stimulator of glucagon secretion [34, 35], which further enhances lipolysis and ketogenesis. Decreased reabsorption of ketones also contributes to ketonemia [36]. Any other precipitants like increased insulin resistance due to stress, extended fasting, or ambitious decrease in insulin secretagogue or insulin could transform the patient from this drug-induced ketogenic state to ketoacidosis [7, 30]. Although there is no established phenotype in type 2 diabetes concerning SGLT-2 inhibitor-associated EDKA, it appears that those with poorer *β* cell function reserve [37], longer duration of diabetes, poorer control of diabetes, and lower BMI are more susceptible to EDKA [38], not to mention the type 1 diabetes [39]. Off-label use of SGLT-2 inhibitors in type 1 diabetes should be taken very cautiously, and some recommended daily monitoring of ketones in the blood or urine [7], which is hard to carry out in the real world (the mechanism of SGLT-2 inhibitor-associated EDKA is illustrated in Figure 2).

One illuminating part of SGLT-2 inhibitor-induced EDKA is that it is more of a “starvation” one than a “diabetic” one. There used to be much emphasis on the distinction between starvation ketoacidosis and EDKA asserting that the latter distinctively results from severe insulin deficiency [24, 40]. SGLT-2 inhibitor-associated EDKA challenges this guardian meaning of being “diabetic.” Interestingly enough, some true EDKA was actually a “starvation” one that happened in a diabetic population [12]. It is not irrational to say that the differential is somehow of nomenclature and is conditional. The SGLT-2 inhibitor-associated DKA is one illustration of the significance of “starvation” in EDKA, just as the fact that starvation rarely causes severe ketoacidosis in a nondiabetic population is an indication of other contributors like insulin deficiency to EDKA.

**Figure 3:**
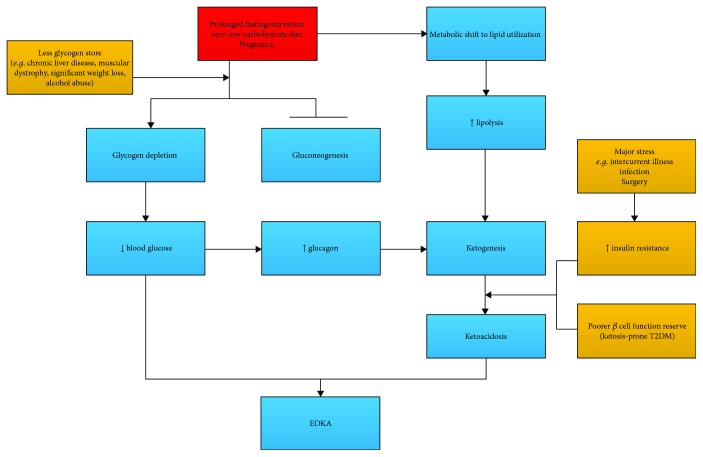
EDKA in a non-insulin-dependent setting: triggered by starvation or other precipitants; with the compensation capacity of EGP impaired by lack of substrates or poor liver function, a metabolic shift to lipid utilization occurs at a lower blood glucose concentration.

**Figure 4:**
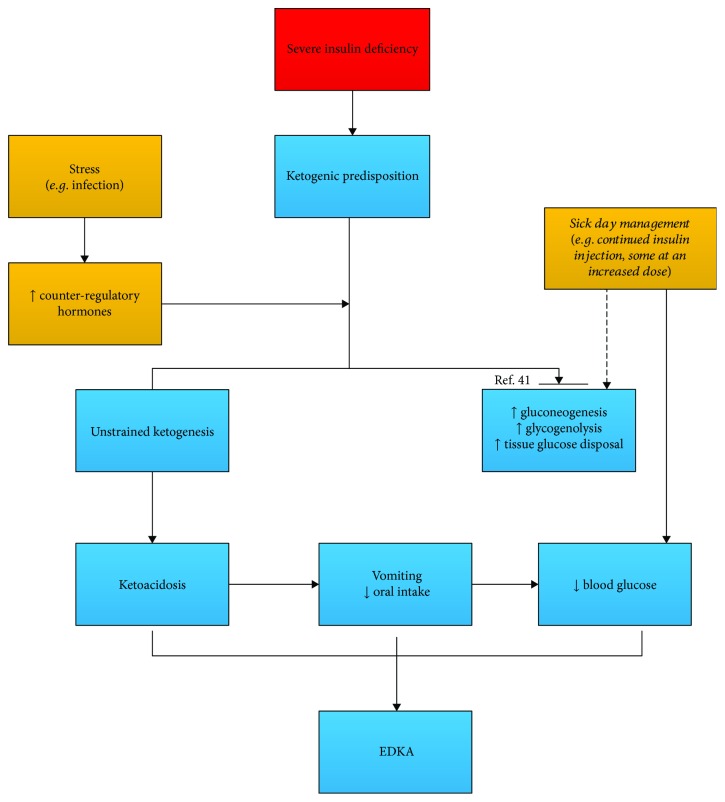
EDKA in an insulin-dependent setting: the hormonal contribution may differ from a non-insulin-dependent setting. With glucagon devoting primarily to ketogenesis (rather than to hyperglycemia) and the remedial insulin treatment, DKA supervenes with a lower blood glucose.

So ketosis can be initiated either by carbohydrate deficit (fasting/starvation, SGLT-2) or by insulin deficiency. The ensuing prognosis of initial ketosis then depends on other factors like insulin deficiency/compensation, intercurrent illness, pregnancy, and sick-day managements. Separate starting points will help to better demonstrate the pathogenesis of EDKA (as shown in Figures 3 and 4).

Starvation-induced ketosis rarely develops to the stage of severe ketoacidosis in a non-insulin-dependent diabetic patient. Precipitants like muscle dystrophy [12], significant weight loss (sarcopenia) [11], and chronic liver disease [24] aggravate glycogen depletion and curtail gluconeogenesis with less substrates and poor liver function reserve. Glucose deficit outraces the compensatory insulin resistance and increased EGP. The resultant low blood glucose and a metabolic shift to lipid utilization along with insulin deficiency end up to EDKA (EDKA triggered by other precipitants in the non-insulin-dependent setting is briefly demonstrated in Figure 3.)

On the background of severe insulin deficiency, the patient is readily predisposed to ketosis. Insulin resistance and elevated counter-regulatory hormones during periods of stress aggravate insulin deficiency to such extent that it cannot be compensated by sick-day managements (insulin increment, fluid intake, and so on) (EDKA in an insulin-dependent setting is illustrated in Figure 4). The work by Meek et al. [41] might further shed some light on the blood glucose control in DKA. In their study, reversal of hyperglucagonemia by liraglutide or a glucagon-neutralizing antibody does not suppress increased hepatic gluconeogenic expression or improve blood glucose control but does attenuate ketosis in uncontrolled diabetes [41]. They further inferred that glucagon is a “redundant” mechanism in diabetic hyperglycemia but a “constant” contributor to ketogenesis. The mechanism behind this uncoupling of the hyperglycemic activity of glucagon from its ketogenic effect might be of molecular level (the depletion of forkhead box transcription factor 1, FOX-1, in *β* cells). If this is true, it would be a new perspective of hormonal interactions in DKA. In the setting of severe insulin deficiency, glucagon might devote primarily to ketogenesis rather than to gluconeogenesis [41]. When hyperglycemia that resulted from insulin deficiency is partly relieved by exogenous insulin replacement, EDKA happens. With this simplified approach to blood glucose in DKA, the claim that EDKA is a partially treated DKA also seems understandable [42]. It may be the truth in some EDKA in type 1 diabetes where the “euglycemic” and “ketoacidic” condition is a temporal response to insulin treatment.

From this point of view, pregnancy is a rather special risk factor for EDKA. It fits into either pattern [43]. With the fetal placenta as an efficient glucose-consuming tissue, there is a shift to lipid utilization in maternal metabolism. Insulin deficiency is also significantly worsened due to the significant insulin resistance from all those placenta-related hormones and other counter-regulatory hormones. So DKA tends to happen more frequently in pregnancy at lower blood glucose levels [23, 44]. The patient is consistently struggling with “glucose hunger” and insulin insufficiency in pregnancy. Even a brief fasting causes more severe ketosis in pregnancy (the “exaggerated starvation”) [44–46]. EDKA in pregnancy could be more misleading with its atypical ABG changes (compensatory respiratory alkalosis is common in pregnancy) in asymptomatic patients [23, 44]. The persistent ketonuria even with her seemingly normal feeding in our case patient #3 is the best demonstration of her “glucose hunger” and insulin insufficiency, indicating that she was on the verge of ketoacidosis all those times. The subsequent test showed anion gap acidosis.

To sum up, the SGLT-2 inhibitor-associated EDKA provides fresh insights into the mechanism of pathogenesis in EDKA. It helps identify susceptible patients with predisposing characteristics. From a practical perspective, there are still many questions awaiting further clarification; for example, what is the exact mechanism of the prolonged glucosuria and even the recurrent ketoacidosis in some cases after discontinuation of SGLT-2 inhibitors [47–49]? What is the clinical significance of concomitant medications that might have influence on inulin/glucagon secretion like statins and ACEI (angiotensin-converting enzyme inhibitors) with SGLT-2 inhibitors [50]? And from the point of EDKA, a combination of metformin and SGLT-2 inhibitors certainly complicates the situation [51].

## Figures and Tables

**Table 1:** Clinical features of 4 cases of EDKA.

		Blood glucose	Urine ketone	*β*-HDB	PH	TG	Scr	Precipitating factors	Effective remedy
Case 1	Type 1 DM	10.4	3+	/	7.12	0.94	42	Reduced insulin dose, poor appetite, sufficient drinking of water	Insulin along with supply of glucose
Case 2	Type 2 DM (new onset)	9.0	3+	/	7.15	1.71	78	Clozapine, poor food intake	Insulin along with increased intake of carbohydrate
Case 3	Type 2 DM in pregnancy	6.7	4+	4.2	7.31	0.62	45	Pregnancy	Insulin along with supply of glucose
Case 4	Type 2 DM with UGIH	9.7	3+	3.1	7.25	1.50	65	Intercurrent illness, discontinuation of insulin pump therapy	Insulin along with supply of glucose

Blood glucose (mmol/l). *β*-HDB: *β*-hydroxybutyric (mmol/l); TG: plasma triglyceride (mmol/l); Scr: serum creatinine (*μ*mol/l); UGIH: upper gastrointestinal hemorrhage.

## Data Availability

The data used to support the findings of this study are available from the corresponding author upon request.
